# A systematic literature review of pediculosis due to head lice in the Pacific Island Countries and Territories: what country specific research on head lice is needed?

**DOI:** 10.1186/1471-5945-14-11

**Published:** 2014-06-24

**Authors:** Rick Speare, Humpress Harrington, Deon Canyon, Peter D Massey

**Affiliations:** 1 College of Public Health, Medical and Veterinary Sciences, James Cook University, Townsville 4811, Australia; 2 Tropical Health Solutions, 72 Kokoda St, Idalia, Townsville 4811, Australia; 3 Atoifi College of Nursing, Atoifi, Malaita Province, Solomon Islands; 4 Office of Public Health Studies, University of Hawaii at Manoa, 1960 East-West Rd, Biomed Building #T103, Honolulu, HI 96822, USA; 5 Health Protection, Hunter New England Population Health, Tamworth 2340, Australia

**Keywords:** Head lice, Pediculosis, *Pediculus humanus* var *capitis*, Pacific Island Countries and Territories, Systematic literature review, Papua New Guinea, Solomon Islands, French Polynesia

## Abstract

**Background:**

Lack of guidelines on control of pediculosis in the Solomon Islands led to a search for relevant evidence on head lice in the Pacific Island Countries and Territories (PICTs). The aim of this search was to systematically evaluate evidence in the peer reviewed literature on pediculosis due to head lice (*Pediculus humanus* var *capitis*) in the 22 PICTs from the perspective of its value in informing national guidelines and control strategies.

**Methods:**

PubMed, Web of Science, CINAHL and Scopus were searched using the terms (pediculosis OR head lice) AND each of the 22 PICTs individually. PRISMA methodology was used. Exclusion criteria were: i) not on topic; ii) publications on pediculosis not relevant to the country of the particular search; iii) in grey literature.

**Results:**

Of 24 publications identified, only 5 were included. Four related to treatment and one to epidemiology. None contained information relevant to informing national guidelines.

**Conclusions:**

Current local evidence on head lice in the PICTs is minimal and totally inadequate to guide any recommendations for treatment or control. We recommend that local research is required to generate evidence on: i) epidemiology; ii) knowledge, attitudes and practices of health care providers and community members; iii) efficacy of local commercially available pharmaceutical treatments and local customary treatments; iv) acceptability, accessibility and affordability of available treatment strategies; and iv) appropriate control strategies for families, groups and institutions. We also recommend that operational research be done by local researchers based in the PICTs, supported by experienced head lice researchers, using a two way research capacity building model.

## Background

When the local community at Atoifi in the Solomon Islands decided to control head lice, they could find no national guidelines. This raised the important question of whether countries and their residents need local evidence to control pediculosis, which is a global problem due to *Pediculus humanus* var *capitis*[1].

Country-specific data is essential for planning communicable disease control programs, even for pediculosis [2]. For more serious diseases the importance of local data is well established. For example, intestinal parasite control activities have to be informed by country, regional and even locally-specific data collected on a regular basis to determine the local epidemiology and extent of parasitic infections with long-term repeated surveillance to inform strategies as the situation changes [3,4]. Quantitative data can be used in modeling to make disease control more cost-effective; e.g., measles vaccination programs [5] and the HIV Spectrum and Estimation and Projection Package programs [6]. In a similar way country-specific and local data arguably can improve control strategies for pediculosis [2]. The value of local research in informing practice was reinforced by a survey in South Africa carried out by the provincial communicable disease program that showed only children of European and Indian ancestry in a mixed race school had pediculosis [7]. This resulted in targeting of Mpumulanga Province’s health departmental control efforts for pediculosis away from the black African students to the other racial groups. From a communicable disease control perspective evidence needed to control pediculosis can be placed in several categories: biology, epidemiology, impact, diagnosis, treatment, societal context and policies.

Head lice have been a topic of research since before the 20^th^ century. Initial studies focussed on biology, epidemiology and techniques to kill lice with the emphasis from the 1940s shifting to research on efficacy of pharmaceutical treatments [8-10]. The emergence of insecticide resistance to organochlorines (DDT and BHC) in the 1970s [11], and then to permethrin in the 1990s [12], expanded the search for alternative insecticidal therapies, and exploration of the mechanisms of action of chemically defined insecticides [13]. Accompanying this was research on control strategies at the community level, particularly in the UK, using physical removal of head lice [14]. In the 21^st^ century research on treatments expanded to include topical silicon-based oils [15] and oral ivermectin [16,17]. Other research included the psychological effect of pediculosis [18] and beliefs and practices of community members from developed and developing countries [19-21].

Various developed countries have published guidelines for management and control of head lice, largely based on the evidence generated by research. A set of international guidelines provided general guidance across many sectors from government to parents [22]. The importance of local data was emphasised for: 1) understanding the pattern of insecticide resistance against the locally marketed products; 2) epidemiological studies in situations where control is ineffective; 3) formulating local recommendations which recognised local cultural factors. The guidelines emphasised the important role of universities and other research institutions in conducting research across the spectrum of pediculosis in their own country.

**Figure 1:**
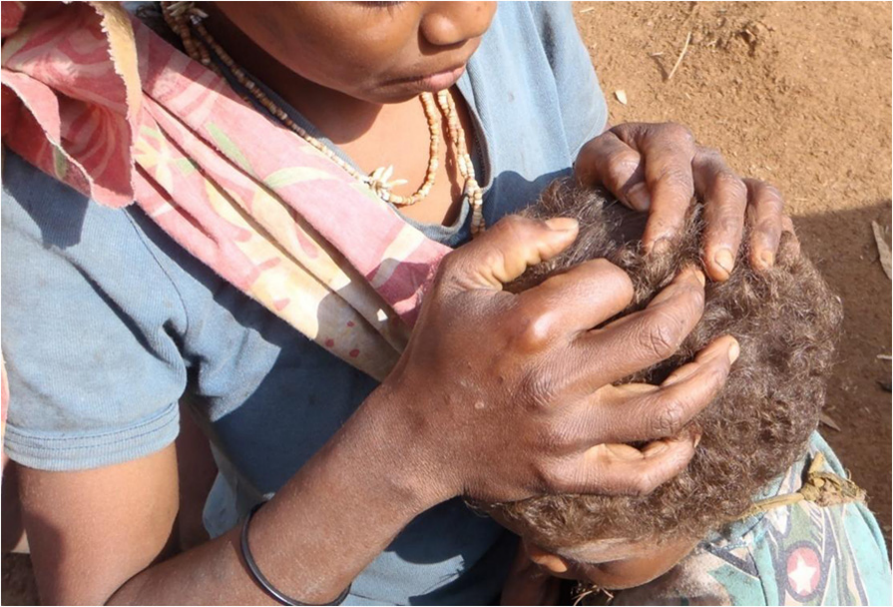
Teenage girl searching for and crushing head lice in a young child (East Kwaio mountains, Malaita Province, Solomon Islands).

Although most head lice research is from a developed country perspective, some of the emerging economies, especially in Latin America, Asia and the Middle East, have begun to publish original research. However, head lice research in the developing countries in Africa and Oceania remains rare [2]. The unspoken assumption seems to be that these regions have bigger and more important issues to deal with than pediculosis.Anecdotal evidence suggests that pediculosis is widespread in the 22 PICTs. However, there appears to be little evidence to support this statement apart from the frequent scene of heads being manually searched and head lice crushed or even eaten (Figure 1).

The biology of head lice (*Pediculus humanus* var *capitis*) is the same globally, but the epidemiology varies with society and cultural behaviour. Feasible treatment options are highly context dependent owing to access to pediculocides, affordability and culturally acceptable behaviour (e.g., head shaving). Insecticide resistance patterns globally are correlated with the use of topical insecticides [23], but these are rarely used in poor societies [20]. Since controlling pediculosis is not a simple task, parents experience difficulty in managing the many aspects of pediculosis control [19,20]. Head lice guidelines that are practical and tailored to fit each groups’ special circumstances can assist parents, often through ensuring that health care providers communicate appropriate and relevant advice.

Pediculosis due to head lice is classified as one of the six Epidermal Parasitic Skin Diseases, an informal subcategory of the Neglected Tropical Diseases [24]. In developing countries, pediculosis appears often to be dismissed as being too minor a problem for health departments faced with managing overwhelming health problems with limited resources. However, the majority of parents and guardians in resource poor countries would arguably prefer feasible options to assist them to manage pediculosis [20].

Since we had difficulty locating evidence to inform local head lice guidelines in the Solomon Islands, we decided to do a systematic literature review of pediculosis in the PICTs. The aim of this review was to evaluate evidence in the peer reviewed literature on pediculosis due to head lice in the 22 nations that form the PICTs.

## Methods

The PRISMA methodology was used to search the peer-reviewed literature [25]. Search terms used were (pediculosis OR head lice) AND the following countries individually: (American Samoa), (Cook Islands), Fiji, (French Polynesia), Guam, Kiribati, (Mariana Islands), (Marshall Islands), Micronesia, Nauru, (New Caledonia), Niue, Palau, (Papua New Guinea), Pitcairn, Samoa, (Solomon Islands), Tokelau, Tonga, Tuvalu, Vanuatu, and Wallis. The following electronic databases were searched between 10-22 August 2013: PubMed, CINAHL, Web of Science, Scopus, and Google Scholar. The reference lists of included papers were subsequently searched for additional papers not found by the database searches.

Inclusion criteria were: the topic of the publication was pediculosis or head lice; the report was about one or more of the 22 PICTs. Exclusion criteria were: i) not on topic; ii) publications on pediculosis not relevant to the country of the particular search even if the publication referred to another PICT since this record was captured under the other PICT; iii) no English title or abstract; iv) in grey literature (i.e., not in a peer reviewed journal or a monograph).

Based on titles irrelevant publications were rejected at country search level. Duplicates from different sources were then collapsed at country level. If content appeared relevant, abstracts were considered, non-relevant articles excluded and reasons for exclusion recorded. Full texts of all remaining publications were obtained and assessed.

The nature of the literature was classified as: i) original research, ii) reviews, iii) program descriptions or iv) commentary/discussion paper using an adapted research identification schema with original research further classified as: (i) descriptive; (ii) measurement study; (iii) operations/intervention research [26].

## Results

**Figure 2:**
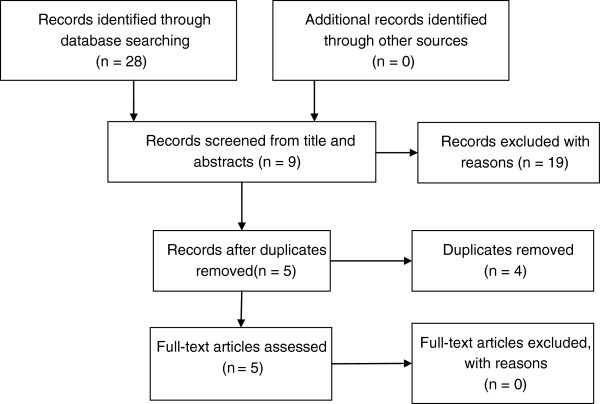
PRISMA flow chart of record retrieval and exclusion.

Five relevant publications were located from an original 28 hits from the database searches (Figure 2). Reasons for rejection of the 19 excluded papers were: i) not on topic = 11; ii) publications on pediculosis not relevant to the country of the particular search = 8.

The five included publications originated from three of the 22 PICTs (French Polynesia, Papua New Guinea (PNG), Solomon Islands) and are summarised in Table 1.

**Table 1:** Publications on head lice or pediculosis in Pacific Island Countries or Territories

**Author (year) citation number**	**Content**	**Type of study**	**Participants**	**Country**	**Classification of study**	**Comment**
Wohlfahrt (1982) [29]	Death with skin ulceration and respiratory failure after self-application of paraquat to head to treat pediculosis	Case report	1 adult male	Papua New Guinea (Western Highlands Province)	Original research; descriptive	No value in informing local guidelines
Eason & Tasman-Jones (1985) [28]	Pediculosis prevalence in Western Province	Cross sectional survey	10,224 total (5,160 < 15 years of age)	Solomon Islands (Western Province)	Original research; measurement	No value in informing local guidelines
Glaziou et al. (1994) [27]	Evaluating the efficacy of oral ivermectin (single oral dose of 0.2 mg/kg) to treat pediculosis	Unblinded single arm therapeutic trial	26 total – 2 males & 24 females aged 5-17 years	French Polynesia	Original research; intervention	Minimal value in informing local guidelines
Thomas (2006) [30]	Ethnomedicine review on use of bark from Galbulimima tree. Mixed with tobacco to treat head lice; no details	Second hand report	No participants	Papua New Guinea (Data collected from Morobe Province)	Commentary	Minimal value in informing local guidelines
Hodgdon et al. (2010) [23]	Genetic study of *kdr* resistance genes in head lice from many countries	Louse genetics	3 lice from 1 person	Papua New Guinea (location not given)	Original research; measurement	No value in informing local guidelines

The only clinical trial was an unblinded single arm therapeutic trial of the efficacy of oral ivermectin in treating pediculosis in French Polynesia [27]. A large cross-sectional survey reported in 1985 examined 10,244 people in the Western Province of Solomon Islands for skin conditions [28]. It found that pediculosis was “universally present among both sexes and all ages”. However, the prevalence of pediculosis was not determined; no additional details were provided. A genetic study on insecticide susceptibility of head lice used a very small sample of 3 lice from PNG [23]. Although it found that insecticide resistance genes were not present in these lice, it was not designed to assess the extent of resistance or decrease in susceptibility (if any) in PNG. Two other studies from PNG dealt with treatment of pediculosis. One was a case report of fatal poisoning from paraquat (a herbicide) misused to self-treat pediculosis [29]. The other was a brief comment that bark of a tree was used in Morobe Province to treat head lice [30]. No evidence on efficacy was provided.

## Discussion

This review found only five publications on pediculosis or head lice from the 22 PICTs. The only epidemiological study was published more than 30 years ago and unfortunately did not quantify the prevalence of pediculosis [28]. Of four papers relevant to treatment of pediculosis, only one was a therapeutic trial [27]. This study demonstrated that lice in French Polynesia could be killed by oral ivermectin with at day 14 elimination of adult lice and nymphs in 100% and 43% of subjects respectively. Side effects were minimal, but the study had important limitations. Another paper found no permethrin resistance (*kdr*) genes in three PNG head lice and the other two treatment papers were case reports. All five reviewed publications have minimal or no value for planning head lice control strategies or informing local guidelines.

One possible limitation to this review is that this systematic review did not include non-peer reviewed literature. However, since the goal was to determine what evidence was available to formulate strategies and guidelines, the focus on peer-reviewed sources would be expected to provide the highest level of evidence available.

If we adopt the perspective of a ministry of health in any country in the PICTs and use the categories of research evidence originally proposed, what country specific data are needed? The international guidelines are useful here, but they recommend further research in all aspects of head lice and their control [22]. The only research topic that is prioritised in the international guidelines is resistance to locally available pharmaceutical products.

We do not consider that research on all aspects of pediculosis control is needed in resource poor countries where research funds and skills are in short supply. The following comments highlight important gaps in understanding in areas where we consider that local research will add useful evidence to guide pediculosis control. Although the comments are targeted towards the PICTs, they possibly have value for other developing countries that do little research on pediculosis.

The biology of head lice is well known and the available knowledge can be assumed to be relevant locally. Research on biology is arguably not required at the country level. Epidemiology is location specific and in the absence of any evidence, making assumptions from another countries’ data is unreliable. The impact of pediculosis on individuals is not well known globally, but presumably there will be lower levels of anxiety in people in the PICTs than in developed countries. There is less need to research this aspect. Diagnostic techniques are well researched and this knowledge can be adopted locally in the PICTs. However, which techniques are feasible is influenced by local socio-economic factors. For example, the baseline diagnostic technique in the PICTs is visual census, with access to nit combs and conditioner being uncommon. Detection of lice by use of conditioner and nit comb is the most sensitive diagnostic technique; however, affordability of and access to conditioner and nit combs in the PICTs make this diagnostic technique inaccessible to most. Research is needed on what diagnostic techniques are feasible and acceptable in the PICTs.Treatment of pediculosis in the PICTs is anecdotally reported to be largely by physical capture of lice (Figure 1), with very limited access to and use of pharmaceuticals. For example, in the Solomon Islands topical 1% permethrin is available from the Ministry of Health by prescription only. Cost of treatment relative to income is a critical factor in choice of therapy and must be considered as evidence in formulating government recommendations, particularly for societies with low average incomes like in the PICTs. Local research on access to and affordability of commercially available treatments is required. Assumptions on the efficacy of treatments based on research evidence from other countries will be unreliable; hence, local research is needed. In addition rural populations have local plant based treatments for pediculosis. These are accessible and inexpensive but local research is needed to test efficacy.

Country specific research on feasible management options for pediculosis is needed. The societal context is highly country specific. How people perceive pediculosis and what they want to do about it determines the feasibility of approaches to management and control [20]. Research is essential on this societal context. Research on effectiveness of policies is also needed. Of course the macro-policies of the ministries of health and education and the micro-policies of individual schools and other institutions should be based on relevant country-specific evidence. However, evaluation of policy interventions is essential and this can only be done by country-specific research. Strengthening the capacity of people within resource poor settings to undertake health research is critical to improving health equity and embedding mutuality throughout the research capacity strengthening process is beneficial [31,32].

## Conclusion

We conclude that there is minimal current local evidence to guide decisions on head lice control in the Pacific Island Countries and Territories. We recommend that basic evidence be generated on: i) epidemiology; ii) knowledge, attitudes and practices of health care providers and community members; iii) efficacy of local commercially available pharmaceutical treatments and local customary treatments; iv) acceptability, accessibility and affordability of available treatment strategies, including non-pharmaceutical measures; and iv) appropriate control strategies for families, groups and institutions. We also recommend that the required research be done by local researchers based in the PICTs, supported by researchers experienced in head lice research, using a two way research capacity strengthening model [30]. The research should have an operational focus to provide results that will assist health decision makers to develop feasible national guidelines and residents of these nations to have access to relevant knowledge and resources to control head lice.

## Competing interests

All authors declare that they have no competing interests.

## Authors’ contributions

RS designed the review, carried out the literature search, assessed retrieved papers and drafted the manuscript. HH designed the review, and assessed the retrieved papers. DC assessed the retrieved papers. PM assessed the retrieved papers. All authors contributed to the final manuscript and all read and approved the final manuscript.

## Pre-publication history

The pre-publication history for this paper can be accessed here:

http://www.biomedcentral.com/1471-5945/14/11/prepub
